# Retraction Note: Pharmacist-driven antimicrobial stewardship program in a long-term care facility by assessment of appropriateness

**DOI:** 10.1038/s41598-021-00221-w

**Published:** 2021-10-12

**Authors:** María Rosa Cantudo-Cuenca, Alberto Jimenez-Morales, Juan Enrique Martínez-de la Plata

**Affiliations:** 1 Pharmacy Department, Hospital Universitario Virgen de las Nieves, Granada, Spain; 2 Pharmacy Department, Hospital de Poniente, El Ejido, Spain; 3 Department of Biochemistry and Molecular Biology II, University of Granada, Granada, Spain

Retraction of: *Scientific Reports* 10.1038/s41598-021-98431-9, published online 23 September 2021

The Authors have retracted this Article.

The intervention described in the Article, as well as some of the primary and secondary outcomes reported, form part of a multicentre clinical trial. The information and data included in this article were published without the permission of the principal investigator, and the experimental design is incorrectly attributed.

All Authors agree with this retraction.

